# Is inflammation a consequence of extracellular hyperosmolarity?

**DOI:** 10.1186/1476-9255-6-21

**Published:** 2009-06-23

**Authors:** Laurent Schwartz, Adeline Guais, Mohammad Pooya, Mohammad Abolhassani

**Affiliations:** 1 Service de Radiothérapie Hôpital Pitié-Salpétrière, Paris, 75013 France; 2 Biorébus, Paris, 75008 France; 3 Pasteur Institute of Iran, BCG Department, Tehran, 13164 Iran

## Abstract

**Background:**

There are several reports suggesting that hyperosmolarity induces inflammation. We recently showed that Dextran Sodium Sulfate causes inflammatory bowel disease due to hyperosmolarity. The aim of this study was to confirm the link between hyperosmolarity and inflammation by assessing osmolarity values in vivo during inflammation, compare the inflammatory potential of different osmotic agents and finally study the long-term consequences of hyperosmolarity on cell fate.

**Methods:**

Osmotic pressures were measured in inflammatory liquids withdrawn from mice subjected to inflammation caused either by subcutaneous injection of Bacille Calmette-Guérin (BCG) or Freund adjuvant. Three epithelial cell lines (HT29, T24 and A549) were exposed up to 48 hours to increasing osmolarities (300, 600, 900 mOsm) of chemically inert molecules such as Mannitol, Propylene Glycol, and Glycerol and inflammatory response was assessed by Enzyme Linked ImmunoSorbent Assay (ELISA) and RNA Protection Assay (RPA). Finally, normal mouse macrophages were exposed to hyperosmotic conditions for long-term culture.

**Results:**

The inflammation caused either by BCG or Freund adjuvant is correlated to hyperosmolarity in inflammatory liquids. The exposure of cells to the different compounds, whatever their molecular weight, has no effect on the secretion of cytokines as long as the osmolarity is below a threshold of 300 mOsm. Higher osmolarities result in the secretion of proinflammatory cytokines (Interleukin-8, Interleukin-6, Interleukin-1β and Tumor Necrosis factor-α). Long-term hyperosmotic culture extends normal macrophage half-life, from 44 days to 102 days, and alters the expression of p53, Bcl-2 and Bax.

**Conclusion:**

The present study further suggests inflammation and hyperosmolarity are closely related phenomena if not synonymous.

## Background

There are scant but reproducible reports in the literature suggesting that hyperosmolarity can cause inflammation [1-8]. We recently published an article showing that any chemical, providing sufficient osmolarity can induce an inflammation [2]. The same osmolarity of chemically unrelated molecules, such as alanine, sodium chloride, or Mannitol, induce similar cytokine secretion.

In epithelial cells, such as normal colorectal epithelial cells, the induction of proinflammatory cytokine secretion through hyperosmolarity is mediated by the methylation of the catalytic subunit of Protein Phosphatase 2A (PP2A) [2]. The methylation of PP2A, in turn, translocates NF-κB a well-known transcription factor, which controls the synthesis of multiple cytokines. Xylitol, a sugar responsible for the methylation of PP2Ac, mimics the inflammatory effect of hyperosmolarity. Okadaic acid, which removes the methyl from PP2Ac, or specific PP2Ac short inhibiting RNAs alleviate the effect of hyperosmolarity [1,2].

The idea that hyperosmolarity regulates intestinal epithelial cell production of inflammatory cytokines is supported by the fact that hyperosmolarity has been reported in several inflammatory bowel diseases. These include Crohn's disease and ulcerative colitis [9,10], as well as the inflammatory bowel disease of the newborn and neonatal necrotizing enterocolitis [11]. In a previous paper [1], we strengthened the link between inflammation and hyperosmolarity, showing that, in vivo, the toxicity of DSS is mediated by hyperosmolarity. Osmolarity of DSS diluted in drinking water below 300 mOsm had no toxic effect while higher osmolarities of DSS caused colonic inflammation. DSS-induced hyperosmolarity in turn results in PP2A methylation, nuclear translocation of NF-κB and the secretion of proinflammatory cytokines by colonic epithelial cells.

The goal of this study is first to measure osmotic pressure in the inflammatory liquids, to generalize the findings that hyperosmolarity can cause inflammation and put forward that hyperosmolarity might have long term consequences on cell fate.

## Methods

### Chemicals

The chemical substances used were the following: D-Mannitol (ref 63650 Fluka), Propylene Glycol (P4347 Sigma) and Glycerol (G2289 Sigma).

### Animals and Acute Inflammation Models

BALB/c mice were obtained from the Centre d'Elevage Janvier (Le Genest, St Isle, France) and maintained in accordance with the European Community's guidelines concerning the care and use of laboratory animals.

BCG-induced acute inflammation was provoked by daily subcutaneous BCG injection (*Mycobacterium bovis *attenuated strain) for one week (n = 6) at three doses (1, 0.1, and 0.01 mg corresponding respectively to 107, 106, and 105 bacilli) [12]. The second model of acute inflammation was induced by Complete Freund Adjuvant (CFA, heat inactivated *Mycobacterium tuberculosis*). CFA was injected in footpad for two days (n = 6) [13]. In both models, inflammatory liquids were withdrawn by introducing an Hamilton's syringe under the skin and osmolarity was measured (blood serum was used as reference) with a cryoscopic osmometer (Osmomat 030, Gonotec, Berlin, Germany).

### Cell culture

The human colon, bladder and pulmonary cancer cell lines, HT-29, T24 and A549 (ATCC numbers HTB-38, HTB-4, and CCL-185, LGC Promochem, Molsheim, France) were cultured in Dulbecco's minimal essential medium (DMEM, Gibco) supplemented with 10% decomplemented fetal bovine serum (FBS, Eurobio, Les Ulis, France) and 1% non-essential amino acids, in a humid atmosphere 5% of CO_2 _at 37°C. Cells were plated at a density of 10^5 ^cells per well in 6 wells-plates 48 hours before treatment. Each well was treated during 24 or 48 hours with one of the 6 compounds of interest, with two different concentrations per compound (corresponding to 600 or 900 mOsm as compared to control 300 mOsm). Osmolarities of the culture medium were measured using a cryoscopic osmometer (Osmomat 030, GONOTEC GmbH, Berlin, Germany). At the end of incubation, supernatants and cells were collected in each plate and pro-inflammatory cytokines were quantified by Enzyme Linked ImmunoSorbent Assay (ELISA) and RNA Protection Assay (RPA).

### Determination of Cytokine Levels

IL-8, IL-6, IL-1β, TNF-α secretions into the supernatants of control or treated HT-29, T24 or A549 cells were quantified by Enzyme Linked ImmunoSorbent Assay (ELISA) by using DuoSet ELISA kits (R&D Systems, Minneapolis, MN), according to the manufacturer's instructions. Colorimetric results were read in an MRX Dynatech Microplate reader (Dynatech, Chantilly, VA) at a wavelength of 450 nm in 96-well high-binding Stripwell Costar EIA microplates (Costar 2592). Substrate Reagent Pack (Catalog # DY999, R&D Systems, Minneapolis, MN) was used for all Streptavidin-HRP reactions. Each sample was assayed in duplicate. For more technical specifications see Abolhassani [2].

### Rnase Protection Assay

RNA was extracted from HT-29 or T24 cells exposed to 900 mOsm during 24 hours or from mouse macrophages exposed to isosmotic or hyperosmotic medium using RNeasy Kit (Qiagen). The mRNA expression was measured by the RiboQuant multiprobe RNase protection assay (BD Biosciences Pharmingen, San Diego, CA). Template RNA probes were transcribed using the cDNA template sets cytokine kits containing either human TNF-α, IL-1β, IL-8 and IL-6 or mouse Bcl-2 and Bax, according to the manufacturer's instructions. For transcription, except for Biotin-16-UTP (Roche Diagnostics, GmbH, Mannheim Germany) all reagents were supplied by the manufacturer.

### PAGE of proteins

Inflammatory liquids were withdrawn from acute inflammation sites and proteins were migrated on a 4.5% polyacrylamide gel electrophoresis (PAGE) gel. The gel was then fixed in a methanol/acetic acid solution and soaked in a silver nitrate solution.

### Macrophage Long-term Hyperosmotic Culture

Bronchoalveolar lavage (BAL) macrophages were obtained from 3 consecutive lavages using 1 ml of 0.9% saline solution each by gentle instillation of BALB/c mice lungs (about 150 mice, 6–7 weeks, female). Purity was controlled by cytology coloration and counting. Cells were cultivated in Dulbecco's minimal essential medium (DMEM, Gibco) supplemented with 10% decomplemented fetal bovine serum (FBS, Eurobio, Les Ulis, France), in the presence of 10 ng/ml M-CSF, penicillin-streptomycin-gentamycin antibiotics and 1% non-essential amino acids, in a humid atmosphere with 5% CO_2 _at 37°C. Hyperosmolarity was obtained by adding Mannitol up to 300 or 600 mOsm to the culture medium. The media were changed every two days.

### Wild-type p53 and Bcl-2 Level Measurement

Total protein fractions from mouse macrophages were extracted and quantified after homogenization at 4°C in RIPA buffer containing 0.1 ml/ml PMSF (SIGMA), 100 μM benzamidine (SIGMA) and 100 mM Na_2_PO_4 _(Prolabo) as protease inhibitors.

Wild type p53 levels were measured by Assay Designs' p53 TiterZyme Enzyme^® ^Immunometric Assay (EIA) kit as described in manufacturer instructions. Protein samples are briefly deposited in a microtiter plate where a monoclonal antibody to p53 is immobilized. After washing, a polyclonal antibody to p53 labeled with the enzyme Horseradish peroxidase (HRP) is added and binds to the p53 protein captured on the plate. After washing out the excess sample and labeled antibody, substrate is added and reacts with HRP-labeled antibody bound to p53 captured on the plate. After a short incubation, the reaction is stopped and the color generated is read at 450 nm.

Wild type Bcl-2 levels were measured by using the same method but with two specific antibodies instead of a commercial kit: monoclonal antibody Bcl-2 (MAB810, Clone 121529, R&D systems) and a polyclonal antibody to Bcl-2 labeled with the enzyme HRP (sc-509, Santa Cruz).

### MTT Tests

Macrophages viability was assessed every day by using a colorimetric method based on the cleavage of the tetrazolium salt, MTT, in the presence of an electron-coupling reagent. Cells, grown in a 96-well tissue culture plate, were incubated with the MTT solution for 4 hours. After this incubation period, a water-insoluble formazan dye was formed. After overnight solubilization, the formazan dye was quantitated using an MRX Dynatech Microplate reader (Dynatech, Chantilly, VA). The absorbance (550 nm) revealed directly correlates to the cell number.

### Statistical Analysis

The non-parametric distribution-free Kruskal-Wallis test was used to compare three or more independent groups of sampled data. When significant differences were found, multiple comparison tests (Tukey test) were carried out to identify which groups are different. Dose dependence of cytokine production was tested by means of ANOVA and regression analysis. All statistical analyses were performed using the GraphPad InStat version 3.0 software for Windows (GraphPad Software Inc., San Diego, CA). Values were considered statistically significant when p was less than 0.05.

## Results

**Figure 1:**
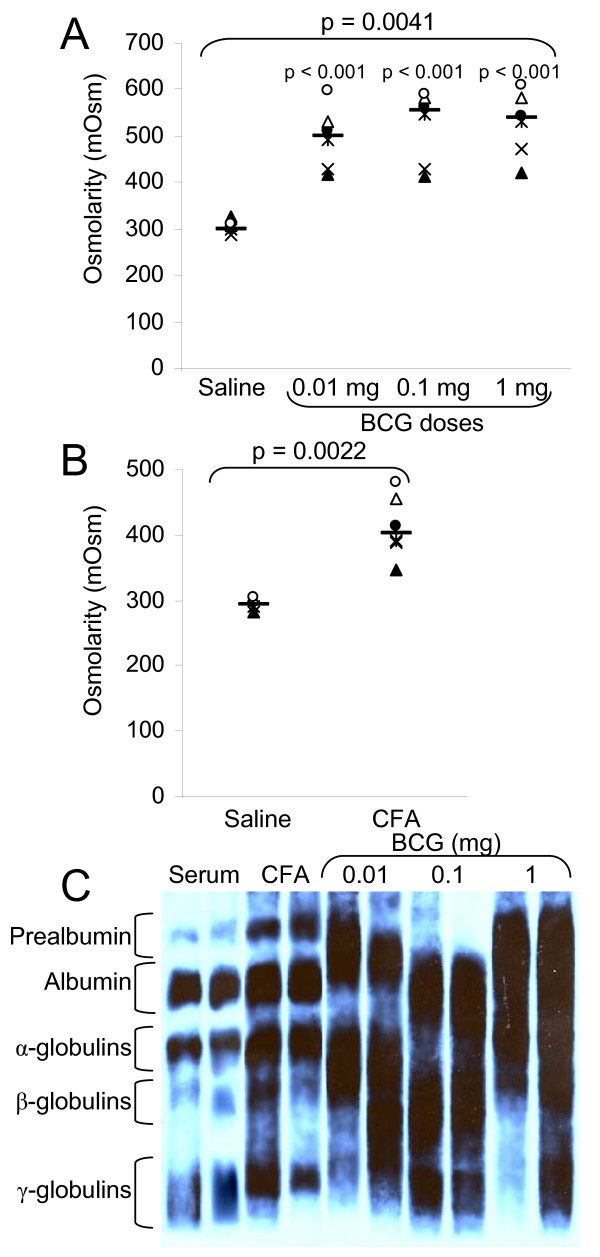
**Osmolarities and protein levels of *in vivo *inflammatory liquids**. Acute inflammation in mice was induced either by BCG (A) (Bacille Calmette-Guérin, *Mycobacterium bovis *attenuated strain) or CFA (Complete Freund Adjuvant) footpad inflammation (B) as described in the Material and Methods section. Inflammatory liquids were withdrawn and osmolarity values measured A) (Saline [291.17–319.06]; BCG 0.01 mg [426.09–567.37]; BCG 0.1 mg [437.07–603.99]; BCG 1 mg [453.22–599.81]) and B) (Saline [282.98–303.08]; CFA [360.90–463.83]). C) Proteins from inflammatory liquids were migrated on a 4.5% PAGE gel and stained with silver nitrate. For each group, the horizontal bar is the median. Upper values represent the global p-values (Kruskal-Wallis test). The p-value given by the Tukey test above a stripchart indicates the result of the comparison of this group versus control. CI at 95% are given in parentheses (n = 6).

The first goal of this study is to measure osmolarity in inflammation caused either by the BCG sub-cutaneous inflammation (Bacille Calmette-Guérin, *Mycobacterium bovis *attenuated strain) and by footpad injection of CFA (Complete Freund Adjuvant, heat inactivated *Mycobacterium tuberculosis*). Both agents cause inflammation at site of injection. This inflammation results in the presence of an inflammatory fluid. In both models, acute inflammation was induced after several daily injections and inflammatory liquids were withdrawn. Osmolarity of these liquids was measured as compared to blood sera and proteins were migrated on a PAGE gel and stained with silver nitrate. Figure 1A shows the osmolarity values of inflammatory liquids induced by BCG. The osmolarity is enhanced from 301 mOsm, in serum, to 526 mOsm, in 1 mg BCG-induced inflammatory liquids. With 1, 0.1, and 0.01 mg of BCG, the average osmolarity values of the inflammatory fluid are respectively 526, 521, and 497 mOsm, and are not significantly different. There is no correlation between the amount of BCG bacilli used to induce the inflammation and the level of hyperosmolarity.

On Figure 1B, the osmolarity values of the extracellular fluid withdrawn after acute CFA-induced inflammation are displayed. The osmolarity is enhanced from 293 mOsm in serum to 412 mOsm. Here again there is hyperosmolarity in the inflammatory liquids.

We then analyzed the protein content of inflammatory liquids (Figure 1C) and found out that inflammatory liquids contained a high level of proteins. These proteins are likely to be responsible, at least in part, of the hyperosmolarity.

**Figure 2:**
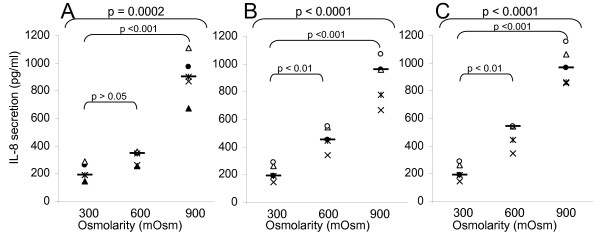
**Interleukin-8 secretion induced by exposure to glycol-derived compounds**. IL-8 secretion (ELISA, in pg/ml) of human T24 cells exposed during 48 hours to increasing osmolarities (300, 600, 900 mOsm) of different compounds: A) Mannitol (300 mOsm [141.23–287.79]; 600 mOsm [246.83–378.69]; 900 mOsm [703.31–1100.9]); B) Propylene Glycol (300 mOsm [141.23–287.79]; 600 mOsm [360.62–568.54]; 900 mOsm [682.24–1084.7]); C) Glycerol (300 mOsm [141.23–287.79]; 600 mOsm [373.93–590.49]; 900 mOsm [816.79–1141.7]). For each group, the horizontal bar is the median. Upper values represent the global p-values (Kruskal-Wallis test). The p-value given by the Tukey test above a stripchart indicates the result of the comparison of this group versus control. CI at 95% are given in parentheses (n = 5).

**Figure 3:**
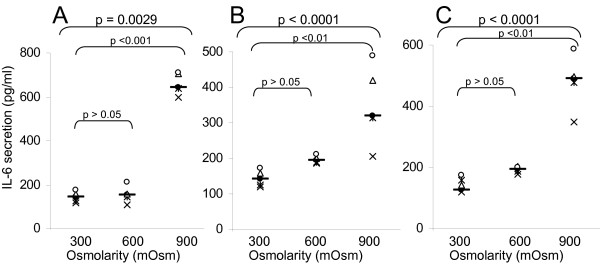
**Interleukin-6 secretion induced by exposure to glycol-derived compounds**. IL-6 secretion (ELISA, in pg/ml) of human T24 cells exposed during 48 hours to increasing osmolarities (300, 600, 900 mOsm) of different compounds: A) Mannitol (300 mOsm [115.47–171.73]; 600 mOsm [108.12–199.79]; 900 mOsm [600.56–716.47]); B) Propylene Glycol (300 mOsm [115.47–171.73]; 600 mOsm [183.87–207.27]; 900 mOsm [214.75–484.27]); C) Glycerol (300 mOsm [115.47–171.73]; 600 mOsm [178.71–205.97]; 900 mOsm [373.47–587.57]). For each group, the horizontal bar is the median. Upper values represent the global p-values (Kruskal-Wallis test). The p-value given by the Tukey test above a stripchart indicates the result of the comparison of this group versus control. CI at 95% are given in parentheses (n = 5).

**Figure 4:**
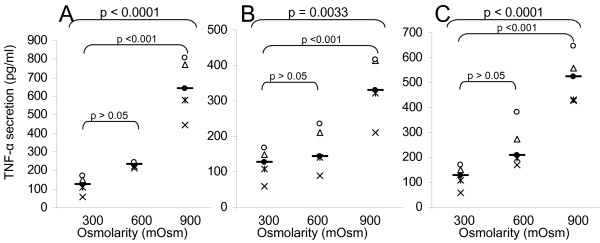
**TNF-α secretion induced by exposure to glycol-derived compounds**. TNF-α secretion (ELISA, in pg/ml) of human T24 cells exposed during 48 hours to increasing osmolarities (300, 600, 900 mOsm) of different compounds: A) Mannitol (300 mOsm [69.40–174.15]; 600 mOsm [214.92–247.70]; 900 mOsm [464.06–829.81]); B) Propylene Glycol (300 mOsm [69.40–174.15]; 600 mOsm [90.364–236.51]; 900 mOsm [232.68–443.57]); C) Glycerol (300 mOsm [69.40–174.15]; 600 mOsm [140.36–351.25]; 900 mOsm [402.68–632.45]). For each group, the horizontal bar is the median. Upper values represent the global p-values (Kruskal-Wallis test). The p-value given by the Tukey test above a stripchart indicates the result of the comparison of this group versus control. CI at 95% are given in parentheses (n = 5).

**Figure 5:**
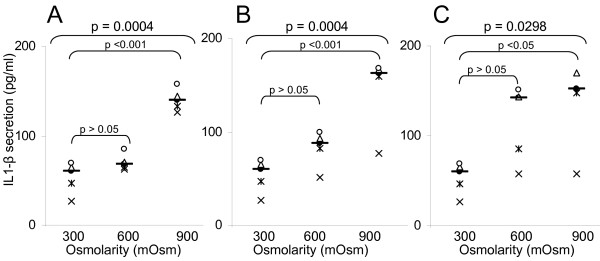
**IL-1β secretion induced by exposure to glycol-derived compounds**. IL-1β secretion (ELISA, in pg/ml) of human T24 cells exposed during 48 hours to increasing osmolarities (300, 600, 900 mOsm) of different compounds: A) Mannitol (300 mOsm [31.969–75.141]; 600 mOsm [59.164–80.941]; 900 mOsm [125.69–154.45]); B) Propylene Glycol (300 mOsm [31.969–75.141]; 600 mOsm [59.674–105.90]; 900 mOsm [97.915–195.67]); C) Glycerol (300 mOsm [31.969–75.141]; 600 mOsm [64.276–167.78]; 900 mOsm [66.748–251.01]). For each group, the horizontal bar is the median. Upper values represent the global p-values (Kruskal-Wallis test). The p-value given by the Tukey test above a stripchart indicates the result of the comparison of this group versus control. CI at 95% are given in parentheses (n = 5).

The second part of the work was to confirm that hyperosmolarity produced by chemically inert molecules could indeed cause inflammation. We performed a large study with glycol-derived compounds in order to assess whether the consequences of hyperosmotic stress observed with other products (cf. previous studies with alanine, Mannitol, NaCl) might be generalized [2]. We cultivated three epithelial cell lines (colorectal HT29, bladder T24, and pulmonary A549) in the presence of rising hyperosmotic concentrations (600 or 900 mOsm) of glycol-derived compounds for 24 or 48 hours and compared their effect on the pro-inflammatory cytokine secretion. The results obtained for the three cell lines are very similar, so, only the data of T24 cells are presented, likewise, only the 48 hours data are presented. Mannitol (Figures 2A, 3A, 4A, 5A) provokes the production of the four pro-inflammatory cytokines tested (p < 0.001 for IL-6, IL-8, TNF-α and IL-1β secretion by T24 cells between 300 and 900mOsm Mannitol). This effect is limited at around 600 mOsm. Propylene Glycol (Figures 2B, 3B, 4B, 5B) and Glycerol (Figures 2C, 3C, 4C, 5C) exhibited the same response profiles (p < 0.001 for IL-8 and TNF-α secretion by T24 cells between 300 and 900 mOsm Propylene Glycol or Glycerol).

**Figure 6:**
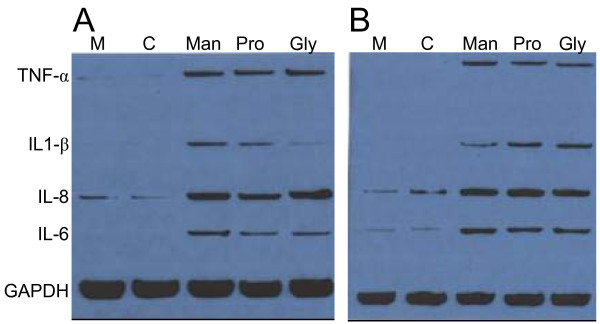
**Pro-inflammatory cytokines transcription following exposure of HT-29 and T24 cells to glycol-derived compounds**. Cytokine transcription was analyzed by RNase Protection Assay performed on RNA from human HT-29 (A) or T24 (B) cells exposed for 24 hours to 900 mOsm Mannitol (Man), Propylene Glycol (Prop) or Glycerol (Gly) media as compared to controls (M: normal medium; C: isosmotic control). Hyperosmolarity increases the level of transcription of IL-8, IL-6, IL-1β and TNF-α. GAPDH transcription is not altered.

To confirm the role of hyperosmolarity in the induction of pro-inflammatory cytokines, we analyzed the RNA production by using an Rnase Protection Assay (RPA). Twenty-four hours after the start of the T24 and HT-29 cells' exposure to 900 mOsm, RNA was prepared for RPA (Figure 6). We observed that the hyperosmolarity induced by Mannitol, Propylene Glycol, and Glycerol raises the different RNA levels of pro-inflammatory cytokine production in a similar manner. In conclusion, whatever the component used, hyperosmotic stimulation induces pro-inflammatory cytokine production.

**Figure 7:**
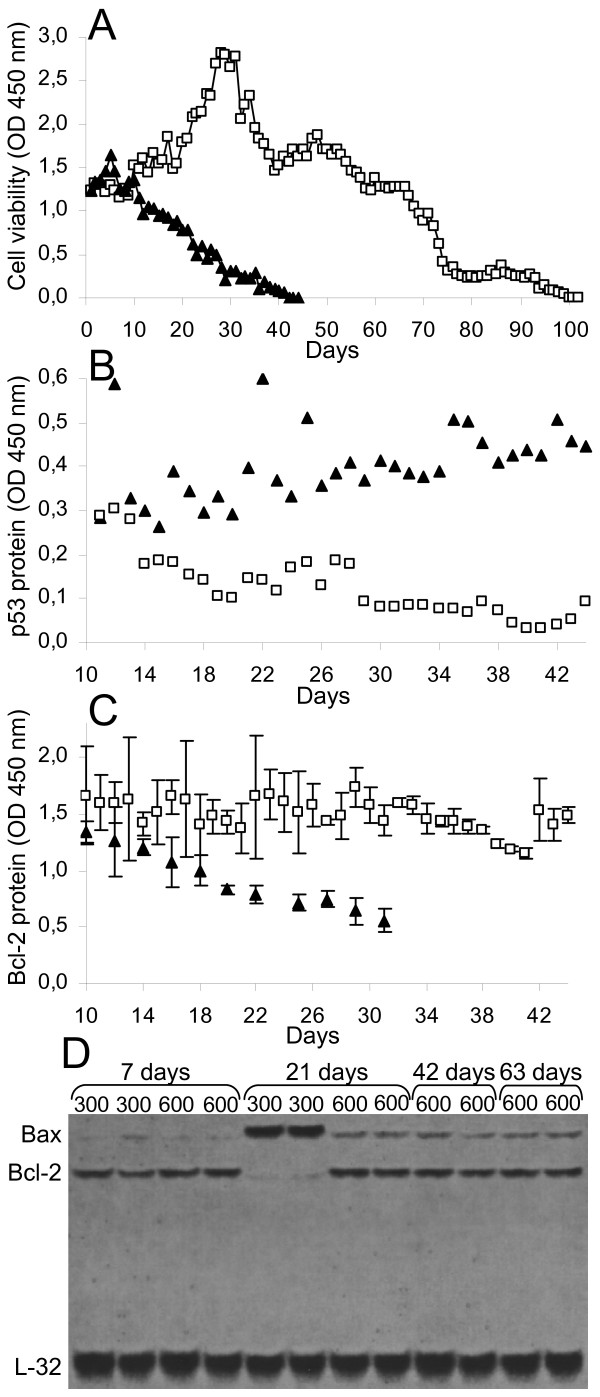
**Long-term hyperosmotic condition culture extends mouse macrophages half-life and alters p53, Bax, and Bcl-2 levels**. BAL macrophages were obtained from 3 consecutive lavages of BALB/c mice lungs. Cells were cultivated in Dulbecco's minimal essential medium in the presence of 10 ng/ml M-CSF. Hyperosmolarity was obtained by adding up to 300 or 600 mOsm Mannitol to the culture medium. Cell viability (OD 450 nm) was assessed every day through an MTT test (A). p53 (B) and Bcl-2 (C) protein expression was measured every other day in whole protein extracts (OD 450 nm) as described in the Materials and Methods section. Rnase protection assay (RPA) of Bax and Bcl-2 was performed (D) (L32 is used as control). Dark triangles represent 300 mOsm cultures and light squares represent 600 mOsm cultures. Data were reproduced in three experiments, each composed of three independent macrophage samples. One representative experiment is presented and each point represents the average of the three samples.

In the third part of our work, we exposed normal macrophages to hyperosmotic conditions for long-term culture. BAL macrophages were prepared and cultivated in hyperosmotic (600 mOsm with Mannitol) versus isosmotic conditions (300 mOsm) for several weeks. Macrophage half-life (Figure 7A) is extended (44 days in isosmotic culture versus 102 days in hyperosmotic culture). By using an MTT test (Figure 7A), we show that cell viability is increased in hyperosmotic conditions. We then analyzed the molecular events that might be responsible for these changes. These macrophages have a modified p53 protein expression as described in figure 7B: p53 level is stable (or maybe slightly higher) during the isosmotic culture whereas the protein is down-regulated in the hyperosmotic culture. Conversely, the Bcl-2 protein level is stable for a long period in hyperosmotic conditions, while it is down-regulated in isosmotic conditions (Figure 7C). These results were confirmed by RPA analysis of Bcl-2 and Bax RNA (Figure 7D). We show an up-regulation of the transcription of the pro-apoptotic gene Bax at day 21 in isosmotic conditions, while it remains constant in hyperosmotic conditions. Conversely, the Bcl-2 anti-apoptotic gene expression remains stable in hyperosmotic conditions until the 63rd day of culture.

In conclusion, long-term hyperosmolarity exposure appears to maintain the low levels of expression of p53 tumor-suppressor protein and Bax pro-apoptotic gene, while it increased the level of Bcl-2 anti-apoptotic gene.

## Discussion

Inflammation is characterized by tumor, dolor, rubor, and calor as stated by Galen two thousand years ago. Inflammation can be caused by factors as diverse as heat, freezing temperature, trauma or multiple chemicals.

In most tissues, with the notable exception of the kidney, the extracellular osmolarity is lower than the osmolarity of the plasma. It is thought to be around 280 mOsm [14]. There are scant data on its evaluation during inflammation [15,16,9,10]. We assessed the osmolarity of the inflammatory fluid in BCG and CFA inflammation models and found an osmolarity of 425–520 mOsm markedly above the normal osmolarity of the extracellular fluid. This increased osmolarity is probably due to protein breakdown [17].

When a foreign body such as a splinter is inserted into the epidermis, there is no inflammation. But, when this splinter reaches the dermis where the capillaries lay, there is inflammation. Vascular leak is a common feature of inflammation. It can be caused by direct damage such as by a foreign body, burn or necrosis. Whatever the cause of the leak, this results in the leakage of red blood cells, leukocytes, and plasmatic proteins. These proteins will, in turn, be broken down into smaller pieces by the metabolic enzymes. This partial digestion will release a larger amount of osmoles, further increasing the extracellular osmolarity. The electrophoresis shown on figure 1 confirms that hypothesis. In the inflammatory fluid caused either by BCG or CFA, there is an increased concentration of proteins.

We have previously shown both in vitro and in vivo, studying the DSS-induced colitis model [1,2], that hyperosmolarity induces pro-inflammatory cytokine responses. Our present work confort the hypothesis that hyperosmolarity and inflammation are closely correlated, if not synonymous. Our "in vivo" results on inflammation caused by BCG and CFA reinforce our findings on DSS; there is a strong correlation between extracellular osmolarity and inflammation. We used three different components. Mannitol is a polyol, similar to Xylitol and Sorbitol. It is a non-permeating molecule, i.e., it cannot cross biological membranes. Mannitol is used clinically to reduce acutely raised intracranial pressure, e.g. after a stroke or head trauma (although significant controversy exists over this use) and to treat patients with renal failure [18]. Propylene Glycol (or propane-1,2-diol) is an organic compound that is hygroscopic and miscible with water [19]. It is widely used as a moisturizer in medicine, cosmetics, food, toothpaste, mouthwash, and tobacco products. Its oral toxicity is low, so it is generally recognized as safe for use as a direct food additive. It is metabolized by the organism into pyruvic acid but does not cross biological membranes, a reason for its use in cryoscopic conservation. Glycerol (or propane-1,2,3-triol) is also used in medical, pharmaceutical, and personal care preparations, providing lubrification as humectant. In organisms, it is a precursor for synthesis of triacylglycerols and phospholipids and it might also enter the pathway of glycolysis or neoglucogenesis. It is used as a cryoprotective agent for cryoscopy as it is a low osmotic agent that slowly crosses the membranes [20]. These three components share the property of inducing hyperosmotic stress, because they do not cross biological membranes and are considered as non-toxic products. We confirm that hyperosmolarity caused by the addition of Mannitol, Propylene Glycol, and Glycerol induces the transcription and the secretion of the following pro-inflammatory cytokines Interleukin-8, Interleukin-6, Interleukin-1β, and Tumor Necrosis factor-α. This stimulation was confirmed using three distinct cancer cell lines.

We cultivated normal macrophages in hyperosmotic conditions in order to evaluate the long-term consequences of hyperosmolarity. It appeared that the 600 mOsm culture extended the macrophage half-life from 44, which is consistent with the duration of macrophage primary culture [21], to 102 days. At the molecular level, it maintained the low levels of expression of p53 and Bax whereas it increased the level of Bcl-2. p53 is a transcription factor situated at the cross-roads of several signaling pathways that are essential for cell-growth regulation and apoptosis [22]. In normal conditions, the level of p53 in the cells is usually limited by a constant degradation of the protein by the ubiquitin/proteasome pathway. When normal mouse macrophages were put in culture in isosmotic conditions, we show that the p53 level is slightly increased until cell death. Consistently, in isosmotic cultures, Bcl-2, a pro-survival protein is down-regulated while Bax, a pro-apoptotic gene transactivated by p53, is overexpressed [23]. However, hyperosmolarity prevents p53 and Bax regulation as well as Bcl-2 inhibition. This suggests that hyperosmolarity prevents apoptosis and allows macrophage half-life to be extended. These might be the initial events in a cell transformation process. During inflammation, there is an influx of macrophages and a high rate of epithelial cell death. Our data strongly suggest that hyperosmolarity appears to play a key role both in the recruitment and the survival of macrophages. But, during inflammation, there is also a massive cell death. Burg has reported that epithelial cultured cell lines undergo apoptosis in response to various levels of hyperosmolarity [for review, see [24]]. But, to our knowledge, no data have ever been reported on normal macrophages in primary culture.

*In vivo*, inflammation is characterized by the production of inflammatory mediators that permit the recruitment of leukocytes, mainly mononuclear cells such as neutrophils and macrophages. Here, we show that hyperosmolarity such as measured in the inflammatory fluid (600 mOsm) more than double macrophage half-life. Thus, hyperosmolarity might be an important survival factor for macrophages that are present in the inflammation site.

The consequences of hyperosmolarity may not be limited to inflammation: first, we know that chronic infection and inflammation contribute to about 25% of all cancer cases worldwide [25]; second, hyperosmolarity has also been measured in tumors [26]. This increased oncotic pressure (osmotic pressure exerted by proteins in plasma) has been mostly studied because it prevents the delivery on target of anti neoplastic drugs. Distribution of antineoplastic agents within tumors remains one of the major challenges in cancer chemotherapy because distribution is hampered by high interstitial oncotic pressure [27,28]. It is possible that cancer might also be a consequence of the massive influx of protein leaked first during chronic inflammation and then by the resulting abnormal blood vessels. The resulting increased pressure may play a major role in cancer development. This hypothesis is reinforced by the fact that we show that hyperosmolarity down-regulates the tumor suppressor gene p53 and delays apoptosis.

## Competing interests

The authors declare that they have no competing interests.

## Authors' contributions

LS designed, performed the research and wrote the paper, AG analyzed the data and wrote the paper, MP performed the research, MA designed, performed the research and analyzed the data.
